# Severe pneumonia caused by human adenovirus type 55 in children

**DOI:** 10.3389/fped.2022.1002052

**Published:** 2022-10-13

**Authors:** Dongwei Zhang, Yi Chen, Tingting Shi, Huifeng Fan, Xingui Tian, Rong Zhou, Li Huang, Diyuan Yang, Gen Lu

**Affiliations:** ^1^ Department of Respiratory, Guangzhou Women and Children’s Medical Center, Guangzhou Medical University, Guangzhou, China; ^2^ Center Laboratory, Guangzhou Women and Children’s Medical Center, Guangzhou Medical University, Guangzhou, China; ^3^ State Key Laboratory of Respiratory Diseases, Guangzhou Medical University, Guangzhou, China; ^4^ Pediatric Intensive Care Unit, Guangzhou Women and Children’s Medical Center, Guangzhou Medical University, Guangzhou, China

**Keywords:** human adenovirus type 55, severe pneumonia, critical pneumonia, clinical feature, children

## Abstract

**Background:**

Emerging human adenovirus type 55 (HAdV-55) causes fatal pneumonia in adults. There is a lack of studies on severe pneumonia caused by HAdV-55 in children.

**Methods:**

We conducted a retrospective review of pediatric patients hospitalized at Guangzhou Women and Children’s Medical Center with severe pneumonia from 2013 to 2020 who had human adenovirus (HAdV) detected in throat samples or bronchoalveolar lavage fluid using RT-PCR. The presence of HAdV-55 was determined by PCR amplification of the hypervariable regions of the hexon gene. Demographic, clinical, etiological, and outcome data were collected and analyzed.

**Results:**

Over the eight-year period, HAdV-55 was detected in three severe and six critical pediatric pneumonia patients. None of the patients had any underlying diseases, and had a median age of 18 months (range, 6–108 months). The male to female ratio was 2:1. All patients presented with fever and cough, and three patients presented with wheezing and diarrhea. Six patients had coinfections with other respiratory pathogens, such as bacteria, Mycoplasma pneumoniae and fungi. Three critical patients developed plastic bronchitis (PB). The median lengths of invasive mechanical ventilation and hospital stay of the critical patients were 10 (8, 28.75) days and 25 (13, 32.25) days, respectively. Three critical patients died, although two of them received extracorporeal membrane oxygenation (ECMO) and blood purification. Three surviving patients developed post-infectious bronchiolitis obliterans (PIBO) at the follow-up.

**Conclusions:**

HAdV-55 can cause fatal pneumonia in children, and shows a high rate of co-infection with other respiratory pathogens and a poorer prognosis combined with PB. Thus, HAdV-55 may be an important subtype in patients with HAdV-induced pneumonia who develop PIBO.

## Introduction

Human adenovirus (HAdV) is an important pathogen causing community-acquired pneumonia (CAP) in children, accounting for 4%–10% of hospitalized cases (1). With the development of phylogenetic analyses based on complete genomic sequences, novel HAdV types have become increasingly identified and characterized. To date, over 100 HAdV genotypes, classified into seven groups (A–G), have been identified (HAdV Working Group, http://hadvwg.gmu.edu/). Among them, HAdV-3 and HAdV-7 most frequently lead to severe pneumonia in children (2, 3). Inparticular, previous research found that Human adenovirus type 55 (HAdV-55) is emerging as a highly virulent pathogen causing acute fatal adenoviral pneumonia among immunocompetent adults (4, 5). HAdV-55, a variant with a recombination of the hexon gene between HAdV-11 and HAdV-14 strains, has also been reported to be associated with multiple outbreaks of severe respiratory tract infections, mostly occurring in students’ training bases, schools, households, and hospitals (6–9). Different HAdV types have different degrees of infectivity and virulence among children and adults. Although HAdV-55 is not the predominant serotype of adenovirus pneumonia in children in contrast to HAdV-7 and HAdV-3, it can still cause severe or critical pneumonia (3, 10). Indeed, HAdV-55 accounts for 3.1%–6.9% of the hospitalized pediatric patients with acute respiratory disease due to HAdV (3, 10, 11). Besides, current knowledge of HAdV-55-induced severe pneumonia in children is relatively rare. Here we describe the clinical features and outcomes of severe and critical HAdV-55 pneumonia in pediatric patients.

## Material and methods

### Study population

We retrospectively collected data of pediatric patients hospitalized at Guangzhou Women and Children’s Medical Center with HAdV-55 induced severe pneumonia between 2013 and 2020.

### Clinical data collection

Clinical information was collected using a standardized data form, and included demographic characteristics (age and sex), comorbidities, clinical symptoms (fever, cough, sputum, dyspnea, chest pain, rash, nausea, vomiting, abdominal pain, diarrhea, and headache), signs (body temperature, heart rate, respiratory frequency, blood pressure, and crackles in the lungs), laboratory tests (whole-blood cell count and blood chemistry), as well as lung microbiological characterization and imaging (computed tomography). Concomitant medications, respiratory support, complications, and outcomes were registered.

Children with respiratory distress and hypoxemia [sustained saturation of peripheral oxygen (SpO2) <90% at sea level] were considered as having severe pneumonia (12). Children considered as having critical pneumonia had ≥1 of the major or ≥2 of the minor following criteria (12): (1) Major criteria: invasive mechanical ventilation; fluid refractory shock; acute need for non-invasive positive pressure ventilation; hypoxemia requiring fraction of inspired oxygen (FiO2) greater than the inspired concentration or flow feasible in general care area; (2) Minor criteria: respiratory rate greater than the World Health Organization normal classification for age; apnea; increased work of breathing (e.g., retractions, dyspnea, nasal flaring, and grunting), PaO2/FiO2 ratio <250, multilobar infiltrates, pediatric early warning score >6, altered mental status, hypotension, presence of effusion, comorbid conditions (e.g., hemoglobin SS disease, immunosuppression, and immunodeficiency); and unexplained metabolic acidosis.

### Sample collection and molecular typing with PCR

Throat swab samples or bronchoalveolar lavage fluid (BAF) were collected from hospitalized pediatric patients with severe or critical pneumonia. The samples were collected and refrigerated at 2–8 °C in a viral transport medium before being transported on ice and analyzed immediately, or stored at −80 °C until analysis. Viral genomic DNA was extracted using a TaKaRa Mini BEST Viral RNA/DNA Extraction Kit Ver.5.0 (TaKaRa, Dalian, China), according to the manufacturer’s instructions and then tested for HAdV using the TaqMan real-time PCR kit (Guangzhou HuYanSuo Medical Technology Co., Ltd., Guangzhou, China) as previously reported (13). HAdV-positive samples were further characterized at the molecular level by PCR amplification of the hypervariable regions of the hexon gene (14, 15). Mycoplasma pneumoniae and respiratory viruses detected by a real-time PCR assay on throat swab sample or BAF. Bacteria and fungi detected by blood, sputum, BAF or bone marrow culture.

### Statistical analysis

Non-continuous variables were summarized as median (interquartile range).

## Results

### Demographics

Over the eight-year period, a total of nine patients with HAdV-55-induced severe pneumonia were admitted to our hospital. All nine patients had a median age of 18 months (range, 6–108 months) and no underlying disease at the time of admission. Five patients were younger than 2 years of age, and four patients were older than 3 years. The male to female ratio was 2:1. Three patients were hospitalized in spring, four in summer, and two in autumn. Five patients were hospitalized in 2019.

### Clinical and laboratory characteristics

The clinical characteristics of the patients are summarized in Table 1. All patients had a disease duration >10 days before admission, either because the condition recurred after discharge or the treatment was not satisfactory at the local hospital before they were transferred to our center. All patients presented with cough and mild-to-high fever. Three patients presented with wheezing and diarrhea. Three patients presented with wheezing and moist crackles on lung auscultation.

**Table 1:** The clinical characteristics of human adenovirus type 55 infection in severe and critical pediatric pneumonia patients.

	Age (months)	Sex	Admission time (season)	Chief complaint	Length of hospital stay (days)	Coinfection
**Critical pneumonia patients**						
Case 1	13	Male	Autumn	Cough and wheezing for 12 days, fever for 8 days	25	MP Escherichia coli
Case 2	60	Male	Summer	Repeated fever and cough for more than 20 days, shortness of breath for 4 days	28	MP Acinetobacter baumannii Pseudomonas aeruginosa Aspergillus fumigatus
Case 3	108	Male	Summer	Repeated fever for 14 days and cough for 7 days	13	–
Case 4	45	Male	Autumn	Repeated fever for more than 15 days	45	Sphingomonas paucimobilis Candida tropicalis Talaromyces marneffei
Case 5	63	Female	Spring	Repeated fever for more than 20 days, cough for 10 days	25	MRSA
Case 6	7	Female	Spring	Repeated cough and shortness of breath for more 15 days	13	HRV
**Severe pneumonia patients**						
Case 7	18	Male	Spring	Repeated cough for more than 2 months, fever for 5 days	12	–
Case 8	12	Female	Summer	Cough for more than 20 days, fever for 6 days	12	MP
Case 9	6	Male	Summer	Cough for 1 month, fever for 2 days	26	–

MP, mycoplasma pneumoniae; MRSA, methicillin-resistant staphylococcus aureus; HRV, human rhinovirus; -, none.

According to the determination criteria, patients 1–6 had critical pneumonia and patients 7–9 had severe pneumonia. Patients with severe pneumonia included two males and one female, with a median age of 12 months (range, 6–18 months). Patients with critical pneumonia included four males and two females, with a median age of 52.5 months (range, 7–108 months). Four critical patients were older than three years of age. The patients’ laboratory characteristics are shown in Table 2.

**Table 2:** The laboratory characteristics of human adenovirus type 55 infection in severe and critical pediatric pneumonia patients on admission.

	Severe pneumonia patients (*n* = 3) median (interquartile range)	Critical pneumonia patients (*n* = 6) median (interquartile range)
**Blood routine (normal range)**		
White blood counts (5–12 10^9^/L)	12.20 (8.50, 14.40)	2.85 (2.23, 6.90)
Neutrophil percentage (40%–60%)	44.00 (25.00, 73.00)	56.00 (47.50, 65.50)
Neutrophil count (2.0–7.2 10^9^/L)	3.72 (2.55, 10.51)	1.91 (1.34, 3.58)
Monocyte count (0.05–0.96 10^9^/L)	0.41 (0.31, 1.01)	0.16 (0.09, 0.30)
Lymphocyte count (1.55–4.8 10^9^/L)	4.46 (2.88, 6.73)	0.74 (0.53, 2.80)
Hemoglobin (105–145 g/L)	118.00 (110.00, 133.00)	107.50 (93.50, 114.50)
Platelet (140–44010^9^/L)	413.00 (208.00, 473.00)	181.00 (89.50, 325.25)
**Blood biochemistry (normal range)**		
Procalcitonin (<0.05 ng/ml)	0.45 (0.10, 0.78)	3.36 (0.75, 10.55)
Hypersensitive C-reaction protein (0–6 mg/L)	5.46 (0.40, 27.54)	12.85 (1.60, 71.79)
Erythrocyte sedimentation (0–20 mm/h)	29.00 (2.00, 30.00)	2.50 (2.00, 4.50)
Alanine transaminase (9–50 U/L)	12.00 (10.00, 18.00)	17.00 (14.00, 158.00)
Aspartate aminotransferase (5–60 U/L)	43.00 (41.00, 83.00)	137.00 (65.00, 341.75)
Creatine phosphokinase-MB (0–37 U/L)	22.00 (21.00, 71.00)	30.50 (23.00, 51.00)
Lactic dehydrogenase (159–322 U/L)	557.00 (328.00, 967.00)	1459.50 (605.00, 1863.00)
Creatinine (18–62 umol/L)	20.00 (15.00, 21.00)	24.00 (21.00, 43.75)
Total bilirubin (2–17 umol/L)	2.90 (2.80, 3.90)	4.80 (3.28, 14.33)
Direct bilirubin (0–7 umol/L)	1.20 (0.80, 1.20)	2.25 (1.55, 8.73)
Albumin (40–55 g/L)	35.90 (33.50, 43.70)	29.85 (26.03, 35.78)
**Coagulation indicator (normal range)**		
Prothrombin time (11–15 s)	13.60 (12.50, 13.80)	13.45 (12.70, 17.13)
Activated partial thromboplastin time (28–45 s)	47.50 (45.40, 57.10)	48.70 (36.10, 52.85)
Fibrinogen (2.00–4.00 g/L)	3.29 (3.28, 3.77)	2.65 (1.51, 3.03)
**Immune indicator (normal range)**		
Immune globulin G (3.82–10.58 g/L)	9.27 (4.87, 18.90)	8.62 (7.32, 10.48)
Immune globulin M (0.40–1.28 g/L)	1.05 (1.01, 1.14)	0.67 (0.48, 1.55)
Immune globulin A (0.14–1.14 g/L)	0.31 (0.22, 0.46)	1.02 (0.32, 1.36)
Immune globulin E (0–60 IU/ml)	30.00 (12.00, 57.00)	87.50 (43.25, 195.50)
C3 (0.80–1.50 g/L)	0.91 (0.87, 1.04)	0.58 (0.53, 0.73)
C4 (0.12–0.40 g/L)	0.25 (0.22, 0.31)	0.21 (0.16, 0.25)

### Microbiological investigation

The co-infections of HAdV-55 with other respiratory pathogens is shown in Table 1. Six patients presented co-infections, of which five were critical. Among the six co-infected patients, three were coinfected with one pathogen, and the other three with more than two pathogen types. The co-infection was of bacterial origin in four patients, *Mycoplasma pneumoniae* derived in three patients*,* and induced by fungi with *Aspergillus fumigatus* and *Talaromyces marneffei* in two cases respectively.

### Chest computed tomography findings

All patients underwent chest computed tomography (CT) scan. The chest CT features were mainly patchy shadows (6/9), lung consolidation (6/9), pleural effusion (5/9), and mediastinal and hilar lymph node enlargement (3/9). Ground-glass opacity was observed in one patient. Among the patients with pulmonary consolidation, four had single lobe consolidation and two had multi-lobe consolidation.

### Fiberoptic bronchoscopy findings

Fiberoptic bronchoscopy (FB) and bronchoalveolar lavage were performed in seven patients. Of those, three revealed a whitish rubbery material occluding the right inferior lobar bronchus (patient 2), multi-lobar bronchus (patient 4), and left inferior lobar bronchus (patient 5), which were considered to be associated with plastic bronchitis (PB). One patient was confirmed to have PB, however the plastic cast was removed successfully through lavage and negative pressure suction. In the other two patients, the plastic cast failed to be successfully removed, therefore the patients were clinically considered to have PB, and refractory hypoxemia could not be improved by invasive mechanical ventilation (IMV). None of the patients were able to undergo multiple bronchoscopic alveolar lavages to remove PB because such an operation under IMV would aggravate hypoxia.

### Treatment, outcome and sequelae

Among the three patients with severe pneumonia, two required oxygen support through inhalation and received 2 mg/kg/day methylprednisolone during 3–5 days for persistent wheezing. All three patients received intravenous immunoglobulin (1 g/kg/d for 2 days) to alleviate inflammatory storms. The average length of hospital stay of these patients was 16.67 ± 8.08 days.

Treatments and outcomes for the critically ill patients are shown in Table 3. Intravenous methylprednisolone was administered to two patients. All patients received either intravenous immunoglobulin, antibiotics, or antifungal therapy. All critically ill patients with respiratory failure received IMV for respiratory support, with a median IMV length of 10 (8, 28.75) days. The median length of pediatric intensive care unit (PICU) and hospital stay was 17 (8.25, 32.25) and 25 (13, 32.25) days respectively. Ultimately, three critical patients died, although two of them had received extracorporeal membrane oxygenation (ECMO) and blood purification. The detailed causes of death in these three patients are shown in Table 3.

**Table 3:** Treatment and outcomes for the severe and critical pediatric pneumonia patients with human adenovirus type 55 infection.

Patient	Intravenous glucocorticoids (methylprednisolone)	Intravenous immunoglobulin (dose, course)	Antibiotic (course, days)	Length of IMV (days)	Blood purification (days)	ECMO (days)	Complications	Length of PICU stay (days)	Length of hospital stay (days)	Outcome	Cause of death	Sequelae
**Critical pneumonia patients**												
Case 1	–	1 g/Kg/d, 2d	Piperacillin- sulbactum sodium (14d)	10	–	–	ARDS	20	25	Survival	/	BO
Case 2	2 mg/Kg/d, 3d	1 g/Kg/d, 2d	Erythrocin (12d) Meropenem (7d) Linezolid (7d) Voriconazole (7d)	24	–	–	ARDS Pneumothorax Pleural effusion PB	28	28	Death	ARDS	/
Case 3	–	400 mg/kg/d, 5d	Imipenem (4d) Meropenem (7d) Vancomycin (7d)	9	1	8	ARDS Pleural effusion	9	13	Death	Septic shock ARDS	/
Case 4	repeated use	repeated use	Piperacillin-tazobactam (21d) Voriconazole (14d) Caspofungin (7d)	43	7	22	ARDS Pleural effusion PB	45	45	Death	MODS	/
Case 5	–	400 mg/kg/d, 5d	Cefoperazone/sulbactam (21d) Vancomycin (21d)	10	–	7	ARDS Pleural effusion PB	14	25	Survival	/	–
Case 6	–	400 mg/kg/d, 5d	Cefoperazone/sulbactam (12d)	5	–	–	ARDS	6	13	Survival	/	–
**Severe pneumonia patients**												
Case 7	2 mg/Kg/d, 3d	1 g/Kg/d, 2d	–	–	–	–	–	–	12	Survival	/	BO
Case 8	–	1 g/Kg/d, 2d	Azithromycin (oral, 3d)	–	–	–	–	–	12	Survival	/	
Case 9	2 mg/Kg/d, 3d	1 g/Kg/d, 2d	–	–	–	–	–	–	26	Survival	/	BO

PICU, pediatric intensive care unit; IMV, invasive mechanical ventilation; ECMO, extracorporeal membrane oxygenation; ARDS, acute respiratory distress syndrome; MODS, multiple organ dysfunction syndrome; PB, plastic bronchitis; BO, bronchiolitis obliterans; -, none.

**Figure 1:**
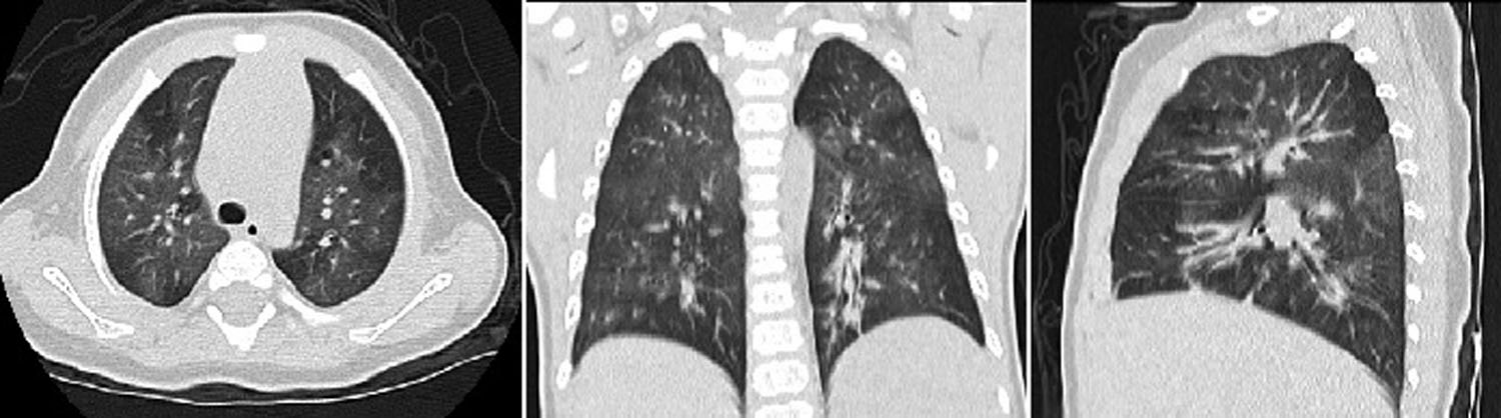
The chest computed tomography of the patient 7 showed mosaic perfusion and air trapping for bronchiolitis obliterans at the follow-up.

Three surviving patients developed post-infectious bronchiolitis obliterans (PIBO) during the follow-up, including two patients with severe pneumonia and one with critical pneumonia. One of them has been symptom-free for 8 years. The other two presented wheezing after physical activity, regularly received inhaled glucocorticoid therapy and have been followed up for 2 years. Chest CT of all patients showed mosaic perfusion and air trapping during follow-up (Figure 1).

## Discussion

The number of children with HAdV-induced CAP has increased significantly due to an epidemic of HAdV infection occurred in Southern China in 2019. During the study period, 55.56% of the patients diagnosed with HAdV-55-induced CAP were infected in 2019. Previous studies have found that HAdV-55 can cause fatal pneumonia in immunocompetent adults (4, 5). Although HAdV-55 can cause severe pediatric pneumonia, it is rarely fatal (3, 10). In the present study, 66.67% of the patients were critical and required PICU admission and IMV. In this regard, although HAdV-3 and HAdV-7 are the most frequent agents causing severe HAdV pneumonia in children, HAdV-55, although less frequently, can also cause severe or fatal pneumonia in children. Thus, pediatricians and PICU staff should recognize HAdV-55 as a potential cause of critical illness.

Epidemiological surveys showed that most children with HAdV pneumonia are younger than 5 years of age, and 50% of the pediatric patients with severe HAdV pneumonia are younger than 2 years of age (10, 16). This may occur because the immune systems of young children are not well-developed, which makes them prone to more severe HAdV disease. In our study, the patients with severe pneumonia were all younger than 2 years of age, however four patients with critical pneumonia were older than 3 years of age. The sample size of this study may be too small to truly reflect the age distribution of severe pneumonia caused by HAdV-55 infection. Meanwhile, HAdV-infected children with underlying diseases are prone to develop severe forms (17). None of the patients included in this study had previous serious underlying diseases such as immunodeficiency, bronchopulmonary dysplasia, premature birth, atopy, or congenital heart disease. In particular, patient 4 was co-infected with *Talaromyces marneffei* which is characteristic of immunocompromised patients, however he was confirmed not to have immunodeficiency by whole gene sequencing. In addition to age, the potential factors leading to critical illness derived from of HAdV-55 are so far undetermined.

The most common clinical manifestations of pediatric HAdV-55-induced pneumonia are fever, cough, and wheezing. However, differences in the relative frequency of symptoms have been reported, such as a higher proportion of patients presenting wheezing and diarrhea in our study than previously published (3). In our study, patients with critical HAdV-55-induced pneumonia manifested shortness of breath, progressive dyspnea, and persistent hypoxia, which were consistent with reported symptoms in adults (18). For patients with severe HAdV-55-induced pneumonia, laboratory tests showed no obvious alterations. However, for critical patients, there were several evident abnormalities in laboratory analyses, such as a decline in white blood cell counts mainly due to a decrease in lymphocytes, increased hypersensitive C-reaction protein and procalcitonin relative to severe cases. Controlled studies showed that the majority of clinical symptoms and signs as well as blood parameters did not significantly differ between HAdV-55 and HAdV-7, with the exception of wheezing, which was higher in patients with HAdV-7-induced pneumonia (3). Concerning laboratory results, in a controlled study of subjects over 7 years of age, the detection rate of procalcitonin (PCT) >1.0 ng/ml, aspartate aminotransferase (AST), and lactate dehydrogenase (LDH) levels were significantly higher in the HAdV-55 group than in the HAdV-7 group, whereas the level of serum albumin was lower. Besides, the HAdV-55 group presented a higher number of severe patients (5). In summary, HAdV-55 is a relevant pathogen causing severe pneumonia in children. A more severe condition is associated with more obvious alterations in laboratory indicators.

Co-infections occurred frequently in our patients. Six patients were co-infected with additional respiratory pathogens. However, when comparing between subgroups, the incidence of co-infection in critical patients was higher than that in severe patients (83.3% vs. 33.3%). Among these patients, three had *Mycoplasma pneumoniae* infection. *M. pneumoniae* is not only the most prevalent organism in co-infection with HAdV, but is also known to easily lead to severe illness when co-infected with HAdV in pediatric patients (19). Other combinations of pathogenic co-infections may have influenced the development of a critical condition in our patients. Some studies have reported that HAdV-55 is associated with other respiratory viral infections (3, 10). However, most of our patients were co-infected with bacteria, *M. pneumoniae*, and fungi. Moreover, two patients who were co-infected with *Aspergillus fumigatus* and *Talaromyces marneffei* died. Complications derived from fungal co-infections do worsen the condition and may be lethal. In this sense, the detection of fungi should be performed as soon as possible for critical patients, as timely and standardized antifungal treatment may improve prognosis.

To our knowledge, pediatric patients with PB secondary to HadV-55 have not been reported. Three critical patients had complications with PB, including one case with a definite diagnosis and two cases with a clinical diagnosis. PB is a rare critical disease characterized by the formation of plastic casts of the bronchial tree that obstruct the airways. HAdV-associated PB is mainly observed in case reports and is caused by HAdV-7 (20–22). Removal of plastic casts through FB is the main treatment for children with PB; however, for critical patients, oxygen support, respiratory support (IMV or non-invasive ventilation), and even ECMO are required (20–22). FB in critically ill patients increases the risk of airway obstruction which may lead to aggravating hypoxia. In fact, 70% of patients with HAdV-associated PB require more than two rounds of FB therapy during hospitalization (20). Two of our patients with PB failed to have their casts removed through FB under IMV and were unable to tolerate prolonged FB along with negative pressure suction. In actual clinical practice, the frequency and length of FB examinations need to consider the patient’s tolerance. Even under the current standard treatment, HAdV combined with PB has a high mortality rate (20). Regarding our patients, two among those with PB who had respiratory failure died. These patients, in addition to respiratory failure, also had high ventilator parameters, prolonged IMV, and hypoxia, which may lead to failure of other organs.

It is generally known that HAdV infection predominates in PIBO, which is a chronic obstruction of the airflow associated with inflammatory lesions of the small airways (16, 23). PIBO is the classical long-term sequela of HAdV infection, mainly observed in HAdV-7 and HAdV-3 infections (24). PIBO associated with HAdV-55 infections is relatively rare. Three patients (33.3%) developed PIBO in our study, consistent with that 10.7%–40% of hospitalized patients with HAdV that developed PIBO according to the literature (16, 25, 26). Consistently, these three patients had risk factors for PIBO, such as persistent wheezing or respiratory failure during a severe pneumonia episode. Thus, HAdV-55 is a relevant subtype in patients with HAdV pneumonia who develop PIBO.

This study had several limitations. First, this was a single-center, small sample study. We did not routinely perform virus typing for mild adenovirus pneumonia. In addition, we did not compare clinical features with those of children with HAdV-7 infection, which is the most common cause of severe pneumonia in children. It is difficult to evaluate the severity of the patient’s condition caused by HAdV-55 or other pathogens due to co-infections. The failure to exclude the influence of co-infection on the results is one of the limitations of this study. Future researchers should adjust the variable of co-infection in the experimental design.

## Conclusion

Our data provides new insights into the clinical features of severe HAdV-55 pneumonia in pediatric patients. HAdV-55 can cause fatal pneumonia in healthy children. Severe, especially critical, patients showed a high rate of co-infection with other respiratory pathogens. Thus, HAdV-55 may be a relevant subtype in patients with HAdV-induced pneumonia who develop PIBO.

## Data Availability

The raw data supporting the conclusions of this article will be made available by the authors, without undue reservation.
